# Frost Work

**Published:** 1877-11

**Authors:** 


### FROST WORK.

Beautiful is it of a winter's morning, to look out upon the snow laden trees, the limbs and twigs bending to the ground with their crystal burden; but there is coldness in that beauty.

Beautiful is it also to gaze upon the sculptured marble, and see its lineaments almost speaking with expression, but that beauty is more than cold, it is dead.

Beautiful is it to gaze upon the *mirage* of the desert, but it is deceitful; and upon the rainbow, and the icicles of a million forms, sparkling like diamonds in the noon days' sun light, but transient, empty, and unreal all.

